# ROS-dependent catalytic mechanism of melatonin metabolism and its application in the measurement of reactive oxygen

**DOI:** 10.3389/fchem.2023.1229199

**Published:** 2024-01-16

**Authors:** Xiangge Tian, Xiaohui Kang, Fei Yan, Lei Feng, Xiaokui Huo, Houli Zhang, Yan Wang, Xia Lv, Xiaochi Ma, Jinsong Yuan, Jiao Peng, Li Dai

**Affiliations:** ^1^ Second Affiliated Hospital, Dalian Medical University, Dalian, China; ^2^ Department of Pharmacy, Peking University Shenzhen Hospital, Shenzhen, China; ^3^ College of Pharmacy, Dalian Medical University, Dalian, China

**Keywords:** melatonin, reactive oxygen species, cancer, CYP450, computational chemistry

## Abstract

Melatonin (Mel) is an endogenous active molecule whose metabolism progress significantly influences its bioactivity. However, the detailed metabolic pathway of Mel in the pathological state has not yet been fully illustrated. In this study, 16 metabolites of Mel in cancer cells and human liver microsomes were identified, of which seven novel metabolites were newly discovered. Among them, 2-hydroxymelatonin (2-O-Mel), as the major metabolite in cancer cells, was revealed for the first time, which was different from the metabolite found in the human liver. Furthermore, CYP1A1/1A2- and reactive oxygen species (ROS)-mediated 2-hydroxylation reactions of Mel were verified to be the two metabolic pathways in the liver and cancer cells, respectively. ROS-dependent formation of 2-O-Mel was the major pathway in cancer cells. Furthermore, the underlying catalytic mechanism of Mel to 2-O-Mel in the presence of ROS was fully elucidated using computational chemistry analysis. Therefore, the generation of 2-O-Mel from Mel could serve as another index for the endogenous reactive oxygen level. Finally, based on the ROS-dependent production of 2-O-Mel, Mel was successfully used for detecting the oxygen-carrying capacity of hemoglobin in human blood. Our investigation further enriched the metabolic pathway of Mel, especially for the ROS-dependent formation of 2-O-Mel that serves as a diagnostic and therapeutic target for the rational use of Mel *in clinics*.

## 1 Introduction

Melatonin (Mel), as an endogenous molecule, was first isolated and identified in the pineal gland. Previous reports revealed the powerful biological functions of Mel (Hardeland et al., 2006). In humans, it acts as a biological modulator and is responsible for the regulation of circadian rhythms through G-protein-coupled melatonin receptors, MT1 and MT2 (Liu et al., 2016). Based on the powerful function and wide distribution of these two receptors, Mel also plays a vital role in the development of various diseases including depression, diabetes, neurodegenerative diseases, and cancer (Sun et al., 2016; Carrascal et al., 2018; Cipolla-Neto and Amaral, 2018; Slominski et al., 2018; Talib, 2018; Cardinali, 2021; Loh and Reiter, 2021). In addition, Mel has anti-inflammatory properties and exhibits some excellent bioactivities such as immune enhancement and the suppression of cancer progression (Tordjman et al., 2017; Talib, 2018).

Mel metabolism is mainly divided into two: enzyme- and no enzyme-dependent metabolism (Tan et al., 2015). The enzyme-dependent metabolism pathway of Mel in humans had been fully illustrated previously (Ma et al., 2005). In humans, the major metabolism pathway is 6-hydroxylation mediated by CYP1A1/2. Following this, the major metabolite 6-OM undergoes sulfation by SULTs including SULT1A1 and SULT1E1 (Ma et al., 2005; Tian et al., 2015). Demethylation is also another major metabolism pathway mediated by CYP2C19 to form NAS, followed by sulfation and glucuronidation reactions. Finally, the major sulfation metabolites (6-OM-S and NAS-S) are excreted through urine (Tian et al., 2015). In addition to the enzyme-dependent metabolism, AFMK is the major metabolite *in vitro* catalyzed by H_2_O_2_ (Tan et al., 2015). However, AFMK is unstable and easily transformed to AMK. Hence, an accurate measurement of AFMK production and investigation of its detailed progress and the underlying mechanism were not possible. Apart from the abovementioned metabolism pathway of Mel, 2-hydroxylation of Mel had also been discovered; however, the detailed mechanism and source had not been fully illustrated. Thus, clarifying the catalytic mechanism and its origin is very helpful for understanding the context and significance of Mel’s metabolism.

Some previous studies suggested Mel as a potential anticancer agent widely used in chemotherapy for different types of cancers, in combination with anticancer drugs (Maroufi et al., 2020; Talib et al., 2021). The significant change of metabolic enzymes in cancer cells makes the metabolism of drugs and endogenous compounds in these cells often different from those in common tissues (Stine et al., 2022). Given Mel’s potential role as an anticancer agent and the different metabolic profiles of cancer cells, this study aims to elucidate the detailed metabolism of Mel in cancer cells. We focus on exploring the potential role of 2-hydroxymelatonin (2-O-Mel), a metabolite of Mel, in cancer cells and investigate the mechanisms behind its production. We hypothesize that reactive oxygen might play a crucial role in this metabolic process and that this might be applied in evaluating the oxygen-carrying capacity of hemoglobin in humans. The findings from this study aim to enrich our understanding of Mel’s metabolism in cancer cells and pave the way for potential diagnostic and therapeutic applications.

## 2 Materials and methods

### 2.1 Materials

Melatonin, N-acetylserotonin (NAS), 2-hydroxymelatonin (2-O-Mel), N^1^-acetyl-5-methoxykynuramine (AMK), 6-hydroxymelatonin (6-OM), and 5-methoxytryptamine (5-MT) were purchased from Sigma-Aldrich (St. Louis, MO, United States). 6-OM-S and NAS-S were the metabolites prepared by Tian et al. (2015). *β*-nicotinamide adenine dinucleotide phosphate disodium salt (NADP^+^), D-glucose-6-phosphate disodium salt (G-6-P), and glucose-6-phosphate dehydrogenase were also obtained from Sigma-Aldrich (St. Louis, MO, USA). Human recombinant CYP450 isoforms including CYP1A1, -1A2, -2A6, -2B6, -2D6, -2C8, -2C9, -2C19, -3A4, -3A5, and -2E1 were purchased from Corning Gentest (NY, United States). In addition, 30% hydrogen peroxide (H_2_O_2_), glutathione (GSH), *α*-naphthoflavone, 8-methoxypsoralen, montelukast, sulfaphenazole, omeprazole, quinidine, clomethiazole, and ketoconazole were purchased from ShanghaiYuan Ye (Shanghai, China). The analysis was conducted using AB Sciex Qtrap-5500 and X500R liquid chromatography mass spectrometers (LC-MS).

### 2.2 The assay incubation system

The assay for the enzyme-dependent hydroxylation of Mel was performed in a standard incubation system with the following components: 100 mM potassium phosphate buffer (KH_2_PO_4_/K_2_HPO_4_, pH 7.4), 1 mM NADP^+^, 10 mM glucose-6-phosphate, glucose-6-phosphate dehydrogenase (final concentration at 1 U/mL), 4 mM MgCl_2_, and metabolic enzymes (0.1 nmol recombinant CYP450 and 0.5 mg/mL HLM), making up the whole volume to 200 μL (Cui et al., 2016). The addition of NADP^+^ initiated enzyme-dependent metabolism progress. Then, 100 μL ice acetonitrile was used for the termination of the reaction and centrifuged at 20,000 g for 20 min at 4°C. Aliquots of supernatants were analyzed using liquid chromatography with tandem mass spectrometry (LC-MS/MS).

### 2.3 Preparation of cell cultures and metabolism of Mel in cancer cells

To fully illustrate the metabolism pathway of Mel in cancer cells, HepG2 cells were seeded in a 6-cm plate at 8.0×10^5^ cells/mL. Mel (final concentration at 100 μM) was added and incubated at 37°C in 5% CO_2_ and 95% air for 48 h. The culture medium and cells were collected and dried in a freeze dryer overnight until all liquids were removed. Finally, the dry residue was dissolved in 200 μL of the mobile phase solution (80% 0.1% formic acid aqueous solution and 20% acetonitrile) and centrifuged at 20,000 g for 20 min, and the supernatant was analyzed by time-of flight mass spectrometry (TOF-MS/MS). In the control group, the blank solvent DMSO replaced Mel, and the other conditions were like the Mel group.

### 2.4 The quantitative analysis of the major metabolites in different cancer cells

In brief, different cells including SHSY-5Y, 7860, CCD, CAR3, H1299, LOSE, LoVo, MCF-7, LO2, U118, RKO, A549, HLF, U87, THP-1, H322, and HepG2 were seeded in the 6-cm plate at 8*10^5^ cells/mL. After the cell attached overnight, the medium was discarded. A fresh medium containing 100 μM Mel was added to the cells. After 48 h, these cells were harvested and resuspended in 200 μL distilled water. The cells were then homogenized by sonication (150 *HZ*, 120 s), and freezing was repeated thrice at −80°C. Subsequently, 100 μL ice acetonitrile was added to the precipitated protein. This was followed by centrifugation at 20,000 g for 20 min. The supernatant was analyzed using LC-MS/MS. Additionally, the culture medium was dried using a freeze dryer. The dry residue was dissolved in a 200 μL mobile phase solution and centrifuged at 20,000 g for 20 min. The supernatant was again analyzed using LC-MS/MS.

### 2.5 The LC-MS/MS method for metabolite analysis

The metabolites of melatonin were detected using an UPLC system (SHIMADZU 30AD), equipped with a binary delivery system, an autosampler, a degasser, and a Kinetex Polar C18 chromatography column (2.1 × 100 mm, 2.6 μm). The mobile phase consisted of 100% acetonitrile (A) and 0.1% formic acid aqueous solution (B) with gradient elution as follows: 0.0–1.5 min, 87% B; 1.50–4.0 min, 87%–85% B; 4.0–5.0 min, 87%–25% B; 5.0–5.5 min, 25%–10% B; 5.5–6.0 min, 10% B; 6.0–6.5 min, 10%–87% B; and 6.5–8.0 min, 87% B. The flow rate was set at 0.3 mL/min, and the injection volume was 4 μL. An Applied Biosystems AB Sciex Qtrap 5,500 mass spectrometer (MS/MS) equipped with an electrospray ionization source was used. In addition, positive and negative scan modes were used simultaneously in this method to analyze the whole metabolites. The temperature was set at 600°C. The air gas CUR flow was 40 L/min; and gases 1 and 2 (nitrogen) were set at 35 and 55 psi, respectively. The optimal *m/z* transition conditions for the metabolites are listed in Supplementary Table S1, according to Tian et al. (2015), Jiang et al. (2016), and Wang et al. (2016).

### 2.6 Enzyme assay to produce 2-O-Mel

The contribution of different CYP450 isoforms for the enzyme-dependent production of 2-O-Mel was evaluated. In brief, in the incubation system (Cui et al., 2016), the recombinant CYP450s, including CYP1A1, -1A2, -2A6, -2B6, -2D6, -2C8, -2C9, -2C19, -3A4, -3A5, and -2E1, were added with the final concentration of CYP450 isoform at 0.1 mg/mL. The Mel concentration was set at 10 μM. The mixed samples were incubated at 37°C for 1 h. Afterward, 100 μL ice acetonitrile was added to terminate the metabolism progress and centrifuged at 20,000 g for 20 min. The supernatant was analyzed using LC-MS/MS for 2-O-Mel production.

### 2.7 Kinetic study and chemical inhibition

An understanding of the metabolism kinetics of drugs mediated by enzymes is important for the rational use of drugs (Tracy and Hummel, 2004; Cui et al., 2016; Tian et al., 2021). Thus, the kinetic for 2-O-Mel production mediated by CYP1A1, -1A2, and HLM was performed. Different concentrations of Mel (0–500 μM) were incubated with CYP1A1, -1A2, and HLM at 37°C for 30 min. Finally, the catalytic velocity of the enzyme was fit into the biphasic kinetics model (Eq. 1) to obtain the kinetic parameters. The biphasic kinetic profile described here has two distinct phases. At low-substrate concentrations, the kinetic profile is curved, as with hyperbolic kinetics; however, at high-substrate concentrations, the velocity of the reaction continues to increase (Tracy and Hummel, 2004).

$$v=\frac{V_{\max1}*S}{K_{m1}+S}+\frac{V_{\max2}*S}{K_{m2}+S}$$ (1)

Next, to confirm the contribution of CYP1As for 2-O-Mel production, a chemical inhibition assay was performed, as in previous reports. In the HLM incubation system, different inhibitors for CYP450 isoforms, including α-naphthoflavone (CYP1A1/2, 1 μM), 8-methoxypsoralen (CYP1A1/2, 5 μM), montelukast (CYP2C8, 2 μM), sulfaphenazole (CYP2C9, 10 μM), quinidine (CYP2D6, 10 μM), clomethiazole (CYP2E1, 10 μM), and ketoconazole (CYP3As, 1 μM), were added and co-incubated with Mel. In the control group, the blank solvent was added instead of inhibitors. The residual activity was calculated by comparing it to the control group, which was considered 100%.

### 2.8 The molecular modeling of Mel with CYP1A1 and CYP1A2

#### 2.8.1 Ligand and protein preparation

For the substrate studied here, melatonin was first optimized at the level of B3LYP (Lee et al., 1988; Becke and Axel, 1992; Becke, 1993)/6–311 + G (2d,p) in Gaussian 16 (Frisch et al., 2016). The partial charges were then assigned using the RESP method based on the local minima geometry converged in the optimization process. Atom types and parameters of the bonds, angles, dihedrals, and van der Waals for melatonin were described with the GAFF force field.

The crystal structures of human cytochromes P450 1A1 (CYP1A1) and P450 1A2 (CYP1A2) were obtained from the PDB database with entry codes 4I8V (Walsh et al., 2013) and 2HI4 (Sansen et al., 2007), respectively. His269 (CYP1A1) was protonated at both δ and ε positions, and Glu466 (CYP1A1) and Glu467 (CYP1A2) were defined in their deprotonated form, according to the calculated pKa values using the PDB2PQR online server. Hydrogen atoms were added, and force field parameters were assigned using the casual force field ff14SB (Tan et al., 2015) in the Amber16 package. Force field parameters for heme group description were collected from the Amber contributed parameters database.

#### 2.8.2 Molecular docking

The AutoDock Vina program and the default parameters were used to obtain series of conformation with melatonin binding to CYP1A1 and 1A2. The exhaustiveness index for searching redundancy was set as 128 to embrace a rather thorough searching space. Vina itself as one of the wide applied score functions, improved to meet a balance for speed and accuracy, was adopted here. Cut-off root mean square deviation (RMSD) for saved conformation was set at 2.0 Å, from which nine conformations of melatonin adopting different binding modes were perverse for an interaction mechanism study.

#### 2.8.3 Interaction mechanism study with molecular dynamics

Each of substrate–enzyme interaction complexes with the intact protein and varied binding conformation of the ligand was first solvated with a truncated-orthorhombic-shaped box, closely resembling the shape of a sphere, in which the solvation layer thickness was at least 12 Å. The TIP3P model, reported by multiple previous studies to work well with the Amber force field, was used to describe the solvation effects. Counterions were then added for system neutrality. In the molecular dynamics (MD) simulation, four cycles of minimization run were applied to gradually release bad contacts. Two cycles of heating procedures were then carried out to gently heat the system to reach 300 K. The 2-ns production stage of the MD process ran under the isothermal−isobaric ensemble condition from which the last 1-ns trajectory was used for energy calculation and interaction analysis. All the MD simulations were executed under periodic boundary conditions (PBCs) with a 2-fs time step. Van der Waals and short-range electrostatic interactions were estimated within a 10-Å cutoff to balance the computational efficiency. The long-range electrostatic interactions were assessed using the particle-mesh Ewald method, and the SHAKE algorithm was applied to all bonds including hydrogen atoms (Kistiakowsky and Williams, 1955). Adoption of the aforementioned setting and parameters for MD studies was chosen based on the conventional simulation procedure and large-scale benchmark studies, aiming for a reasonable and well-performing approach in modeling protein–ligand interaction systems. The final interaction model for each substrate–enzyme interaction system was selected based on the binding energy (enthalpy) calculation.

### 2.9 Reactive oxygen species-dependent generation of 2-O-Mel

Apart from the enzyme-dependent production of 2-O-Mel, reactive oxygen species (ROS) catalytic capability toward the hydroxylation of Mel was evaluated using H_2_O_2_ as the donor of ROS *in vitro*. First, dose-dependent H_2_O_2_ was used to produce 2-O-Mel under 5 μM Mel concentration to obtain the saturation concentration of H_2_O_2_ during the catalytic progress. Next, different concentrations of Mel (0–600 μM) were added to H_2_O_2_ (final concentration at 1 mM) to obtain the affinity of Mel toward H_2_O_2_ for the hydroxylation reaction.

Moreover, to confirm that the production of 2-O-Mel was ROS-dependent, GSH—as a classic ROS depletion reagent—was added to the ROS-dependent reaction with concentrations at 1, 2, 5, and 10 mM. In the control group, the blank solvent was added instead of GSH. Eventually, the production of 2-O-Mel was compared to the control group to ensure the influence of GSH on the ROS-dependent hydroxylation reaction.

### 2.10 The ROS-dependent hydroxylation of Mel in living cells

In the aforementioned study, 2-O-Mel was proved to be a major metabolite in various cancer cells. To clarify that the major contribution was ROS-dependent, an acetaminophen (APAP)-induced cell model was used. Acetaminophen (APAP) is a drug commonly used in clinical settings, which induces oxidative stress. Dose- and time-dependent APAP-induced ROS generation in the HepG2 cell model was established. In brief, different concentrations of APAP such as 0, 2.5, 5, 7.5, and 10 mM Mel (100 μM) were added to HepG2 cells and incubated for 24 h. The culture medium was collected and handled as in the above description (Section 2.3) and analyzed using LC-MS/MS to measure the production of 2-O-Mel. ROS generation was also measured in the parallel group in the absence of Mel using a reactive oxygen species assay kit. The correlation between ROS generation and 2-O-Mel production was analyzed. The time-dependent experiment from 0 to 24 h was also performed, and the Mel concentration was set at 7.5 mM.

### 2.11 The catalytic mechanism exploring for ROS-dependent hydroxylation of Mel

All calculations were performed with the Gaussian 16 program (Frisch et al., 2016). For enzymatic reactions, the oxidized active species were generally mimicked using iron-oxo porphyrin with a thiolate axial ligand Fe_4_^+^O_2_^−^(C_20_N_4_H_12_)^−^ (SH)^−^ (Cpd I) as an ideal model (Hackett et al., 2007; Wang et al., 2007; Trott et al., 2010; Wang et al., 2012). The spin-unrestricted B3LYP functional was utilized with the basis set BSI [LACVP (Fe)/6-31G*(C, H, O, N, S)] for geometry optimization and with the basis set BSII [LACV3P (Fe)/6–311++G**(C, H, O, N, S)] for single-point energy calculations (Lee et al., 1988; Becke, 1993; Meunier et al., 2004; Zhang et al., 2017). Numerous density functional theory (DFT) studies confirmed that Cpd I involves two energetically closely lying spin states, that is, the low-spin (LS/S = 1/2) doublet and high-spin (HS/S = 3/2) quartet states, which originate from two spin-up electrons on the iron-oxo moiety and one spin-up/down electron on the porphyrin part (Hirao and Chuanprasit, 2015; Zhang et al., 2017). For non-enzymatic aqueous environments, we employed the spin-restricted B3LYP functional with the equivalent-level basis sets. Each optimized structure underwent harmonic vibration frequency analysis to characterize a minimum (Nimag = 0) or transition state (Nimag = 1) and to provide thermodynamic data. The transition-state structures, which connected the reactant and product on either side, were followed using the intrinsic reaction coordinate (IRC). In single-point energy calculations, solvation effects were accounted for using the PCM (Barone et al., 1998) solvation model with dielectric constants of ɛ = 5.7 and 78.4 to estimate the polar protein environment and the non-enzymatic aqueous environment, respectively (de Visser and Shaik, 2003; Shaik et al., 2005). The gas-phase single-point energy at the level of (R/U) B3LYP-D3(B3LYP with Grimme’s DFT-D3 correction)/BSII//B3LYP/BSI, with the zero-point energy (ZPE), was denoted as Egas (Grimme, 2006; Lonsdale et al., 2010), while Esol included bulk polarity effects and ZPE corrections (Wang et al., 2012; Zhang et al., 2017).

### 2.12 The application of 2-O-Mel in the measurement of the oxygen-carrying capacity of hemoglobin

Hemoglobin plays a vital role in carrying oxygen in humans. In our present study, fresh blood from 149 healthy humans was obtained from The Second Hospital of Dalian Medical University. The oxygen-carrying capacity of hemoglobin was measured using the reaction of Mel to 2-O-Mel, mediated by reactive oxygen carried by hemoglobin. In brief, Mel was added to 200 μL blood samples with the final 10 μM concentration and incubated at 37°C for 30 min. Then, 100 μL ice acetonitrile was added to terminate the reaction and immediately centrifuged at 20,000 × g for 20 min at 4°C to separate Mel and hemoglobin. The supernatant was analyzed using LC-MS/MS to obtain the production of 2-O-Mel. At last, linear regression was performed between the generation of 2-O-Mel and the concentration of hemoglobin to obtain the correlation coefficient (r-value).

## 3 Result

### 3.1 The identification of Mel metabolites in cancer cells

**FIGURE 1:**
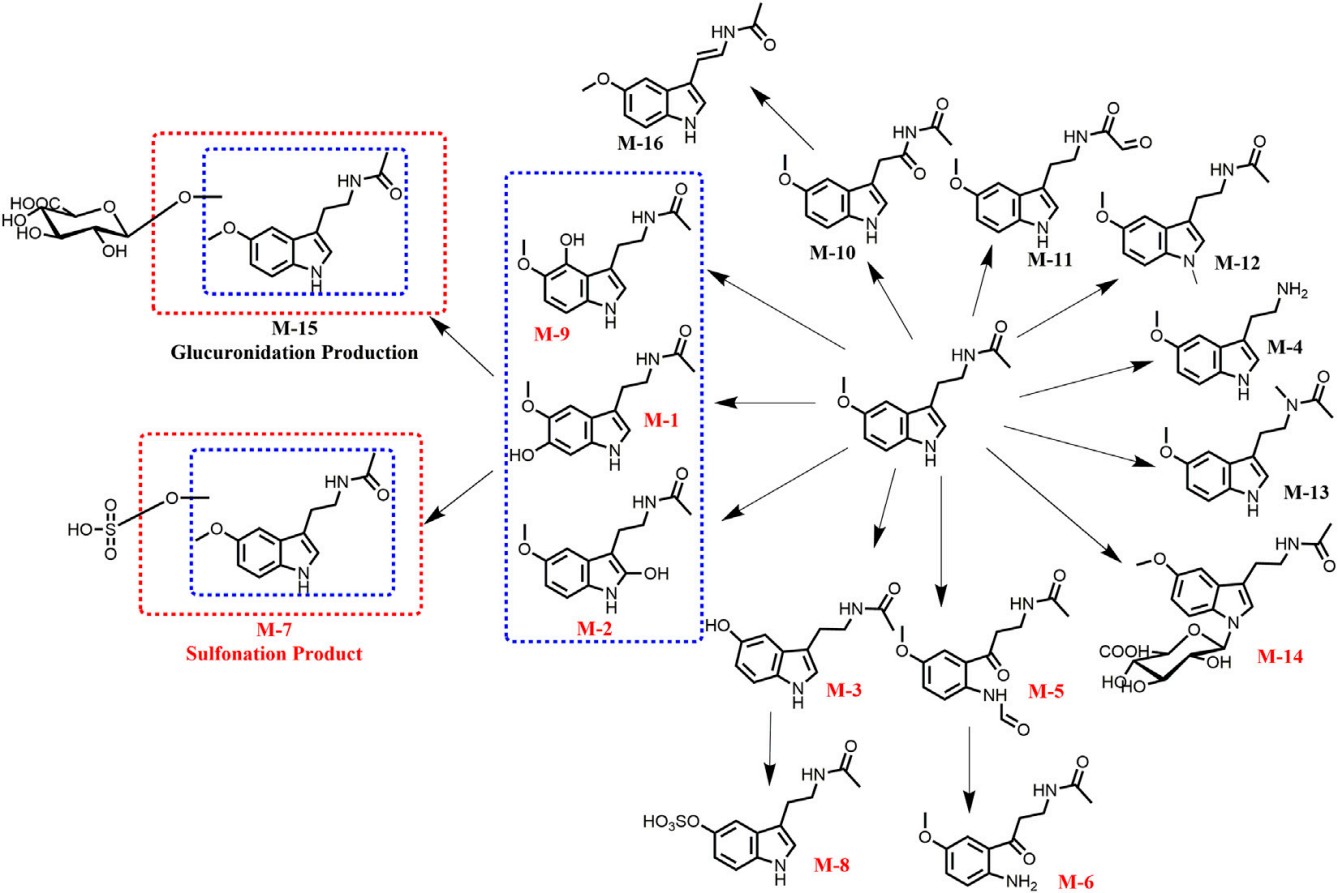
Identification of the melatonin metabolites in HepG2 cells by TOF-MS/MS.

This study identified the metabolic pathway of Mel in HepG2 cells. As illustrated in Figure 1, 16 metabolites (M1–M16) were detected and identified through LC-TOF-MS/MS which was equipped MetabolitePilot 2.0.4 software. Among these metabolites, besides nine previously reported Mel metabolites (red), seven new Mel metabolites (black) were discovered in cancer cells. The mass spectrum fragments of these metabolites are displayed in Supplementary Figures S1–S4. These metabolites represent various metabolic pathways, including oxidation (M-10 and M-11), reduction (M-16), hydrolysis (M-4), and conjugation reactions (M-12, M-13, and M-15).

Some metabolites, such as M-7, M-8, and M-15, are products of further metabolism of Mel’s phase I metabolite. For example, Mel can be metabolized into hydroxylated Mel, which can then be further metabolized by uridine 5′-diphospho-glucuronosyltransferase (UGTs) and sulfotransferases (SULTs). Of these, N-acetylserotonin (NAS, M-3), a major phase I metabolite, can be further metabolized by sulfotransferases (SUTs) to form NAS-S (M-8). This finding aligns with several previous reports (Tian et al., 2015).

### 3.2 The quantitative analysis of major metabolites of Mel in cancer cells

**FIGURE 2:**
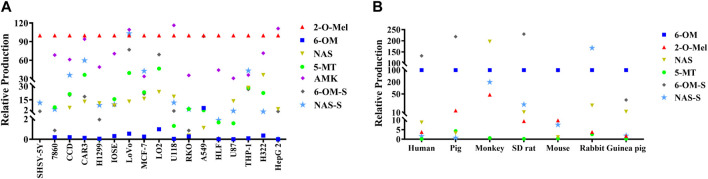
Quantitative analysis of various metabolites of melatonin in various cancer cells **(A)** and liver microsomes from different species **(B)**.

The major metabolites in cancer cells, including 6-hydroxymelatonin (6-OM), 2-hydroxymelatonin (2-O-Mel), N-acetylserotonin (NAS), 5-methoxytryptamine (5-MT), acetylated melatonin (AMK), 6-hydroxy-melatonin-sulfate (6-OM-S), and N-acetylserotonin-sulfate (NAS-S), were quantitatively analyzed (see Supplementary Figure S4). As shown in Figure 2, all major metabolites found in the human liver were also detected in various cancer cells. However, in contrast to the animal liver where 6-OM and 6-OM-S are the predominant metabolites (Figure 2B), a finding is consistent with Ma et al. (2005). The production of 6-OM in various cancer cells was minimal. Instead, the production of 2-O-Mel emerged as the dominant metabolic pathway (Figure 2A). These results highlight a significant divergence in the metabolism between cancer cells and normal liver microsomes, particularly with respect to 2-O-Mel formation. This discrepancy could be attributed to the unique microenvironment within cancer cells. The newly identified 2-O-Mel metabolic pathway in cancer cells warrants further exploration and elucidation.

### 3.3 The enzyme-dependent metabolic pathway for 2-O-Mel

**FIGURE 3:**
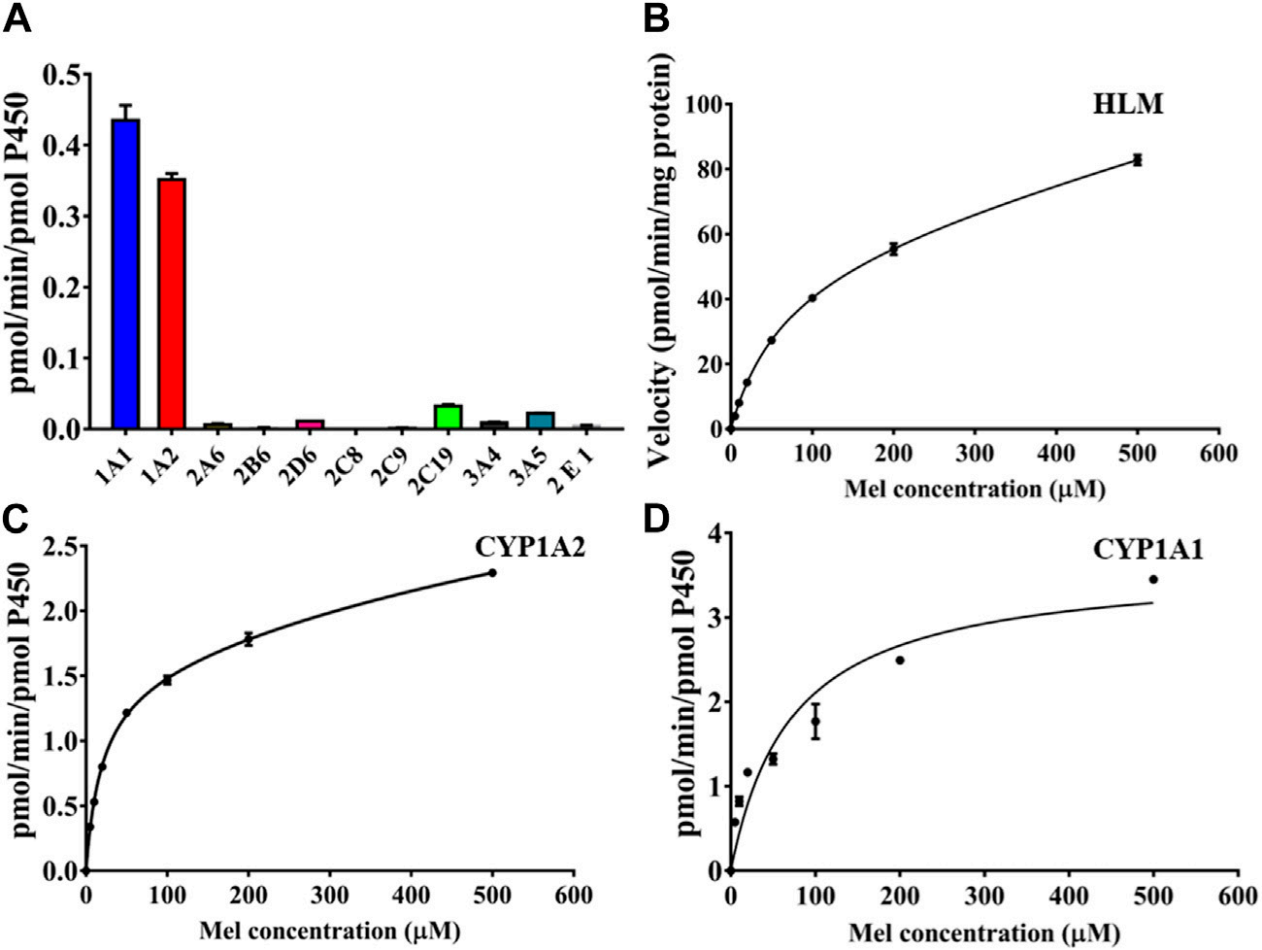
**(A)** Isoform screening for the enzyme-dependent production of 2-O-Mel in various CYP450 isoforms. **(B–D)** Kinetic curves of 2-O-Mel generation in HLM **(B)**, CYP1A2 **(C)**, and CYP1A1 **(D)**.

To elucidate the formation of 2-O-Mel, we screened the catalytic activity of different cytochrome P450 (CYP450) isoforms (Figure 3A). Among various CYP450 isoforms, CYP1A1 and CYP1A2 demonstrated significant activity for the formation of 2-O-Mel, while CYP2C19 and CYP3A5 showed only minimal activity. We then performed metabolism kinetics of melatonin in human liver microsomes (HLMs), as well as in the recombinant CYP1A1 and CYP1A2 isoforms. We observed that the conversion of melatonin to 2-O-Mel followed a biphasic kinetic model in all cases. The kinetic parameters for HLM (V_max_: 56.29 nmol/min/mg, K_m_: 67.19 μM), CYP1A1 (V_max1_: 1.01 nmol/min/pmol CYP, K_m1_: 4.00 μM; V_max2_: 4.23 nmol/min/pmol CYP, K_m2_: 393.5 μM), and CYP1A2 (V_max1_: 1.50 nmol/min/pmol CYP, K_m1_: 19.15 μM; V_max2_: 2.90 nmol/min/pmol CYP, K_m2_: 1,205 μM) were calculated (Figures 3B–D).

**FIGURE 4:**
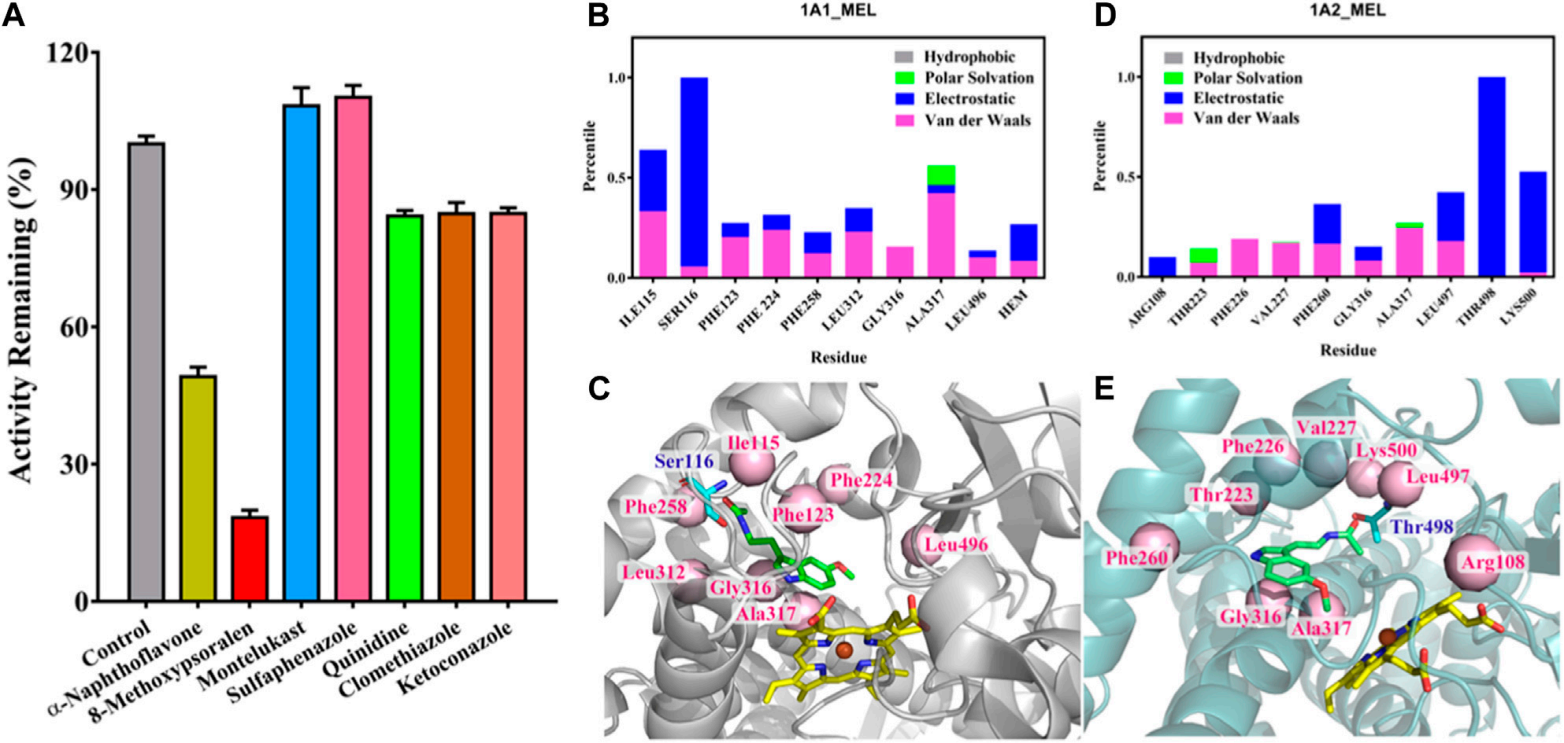
**(A)** Chemical inhibition of 2-O-Mel generation by various CYP450 inhibitors in enzyme-catalytic metabolism progress. **(B–E)** Key residues that remarkably stabilize the substrate–enzyme binding interaction and their contribution format for melatonin with CYP1A1 **(B)** and CYP1A2 **(D)** and the substrate–enzyme interaction of melatonin with CYP1A1**(C)** and CYP1A2 **(E)**.

Next, we used chemical inhibitors to examine the major CYP450 isoform involved in the metabolism of melatonin to 2-O-Mel. Among various CYP450 inhibitors tested, only α-naphthoflavone and 8-methoxypsoralen, inhibitors for CYP1A1/2, significantly reduced the formation of 2-O-Mel (Figure 4A). This reinforces the major roles of CYP1A1 and CYP1A2 in the enzyme-dependent formation of 2-O-Mel in the normal liver metabolism. Lastly, we examined the enzyme-catalytic metabolism process. As shown in Figures 4B–E, the key residues stabilizing the substrate–enzyme binding interaction and their contribution formats were evaluated for melatonin with CYP1A1 (Figures 4B, C) and CYP1A2 (Figures 4D, E).

### 3.4 The catalytic mechanism of Mel mediated by CYP450

We determined the crucial role of CYP1A1 and CYP1A2 in the enzyme-dependent formation of 2-O-Mel. The interaction and catalytic mechanisms of CYP450 during the hydroxylation of melatonin were clarified via molecular docking. Following an energy calculation-based model definition scheme, we first obtained stable interaction models for Mel binding in CYP1A1 and CYP1A2, as indicated by the root-mean-square deviation (RMSD) of the system, reaching ideal equilibrium states (Supplementary Figure S5). Mel’s interaction mechanism in CYP1A1 and CYP1A2 differs. Before catalysis by CYP1A1, Mel displays stable binding facilitated by surrounding residues like Ile115, Ser116, and Ala317, among others (Figures 4B, C). The heme group, especially in the polar format, is also among the top 10 key residues contributing substantially to the Mel–CYP1A1 interaction. The most significant stabilization effect in the Mel–CYP1A2 interaction comes from Thr498 and Lys500 (Figures 4D, E). However, in CYP1A2, the heme group did not significantly aid in Mel recognition and binding stabilization, making Mel less favored in CYP1A2 catalysis. This observation aligns with the reduced turnover rate from kinetic studies when compared with CYP1A1 catalysis.

### 3.5 The ROS-dependent characteristic of 2-O-Mel formation

**FIGURE 5:**
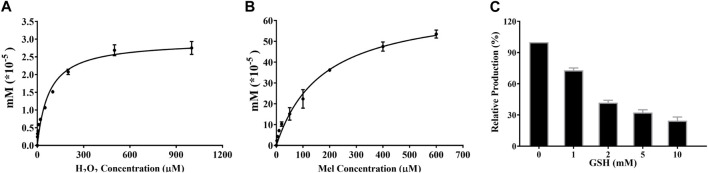
**(A)** Enzyme-independent production of 2-O-Mel under different concentrations of H_2_O_2_. **(B)** Kinetic curve of 2-O-Mel production in the saturated H_2_O_2_ concentration. **(C)** Dose-dependent inhibition of GSH toward 2-O-Mel production.

Free radicals and reactive oxygen species (ROS) are produced in the cells by enzymatic and non-enzymatic reactions, especially in cancer cells that always maintain a high ROS environment (Zhang et al., 2016). To confirm the contribution of ROS in the formation of 2-O-Mel in the cancer cells, an *in vitro* assay was conducted using H_2_O_2_ as a ROS donor. As shown in Figure 5A, with the increase in the H_2_O_2_ concentration (0–1 mM), the formation of 2-O-Mel gradually rises and tends to be stable at 1 mM, complying with the Michaelis–Menten behavior. Next, the formation of 2-O-Mel from the concentration-dependent Mel is also analyzed, as shown in Figure 5B. The formation of 2-O-Mel complies with the Michaelis–Menten behavior, and the obtained K_m_ is 300 μM. Moreover, the formation of 2-O-Mel in the presence of H_2_O_2_ is blocked by GSH (a ROS-scavenger) in a dose-dependent manner (Figure 5C). Previous studies indicated that various CYP450 isoforms exhibited an abnormal expression in the tumor microenvironment, for example, low expression of CYP1A1/2 in cancer cells. The aforementioned results suggested that apart from the enzyme-dependent formation of 2-O-Mel, ROS played a vital role in the metabolism of 2-O-Mel, which is the major metabolic pathway in cancer cells.

### 3.6 The ROS-dependent 2-O-Mel in cancer cells

**FIGURE 6:**
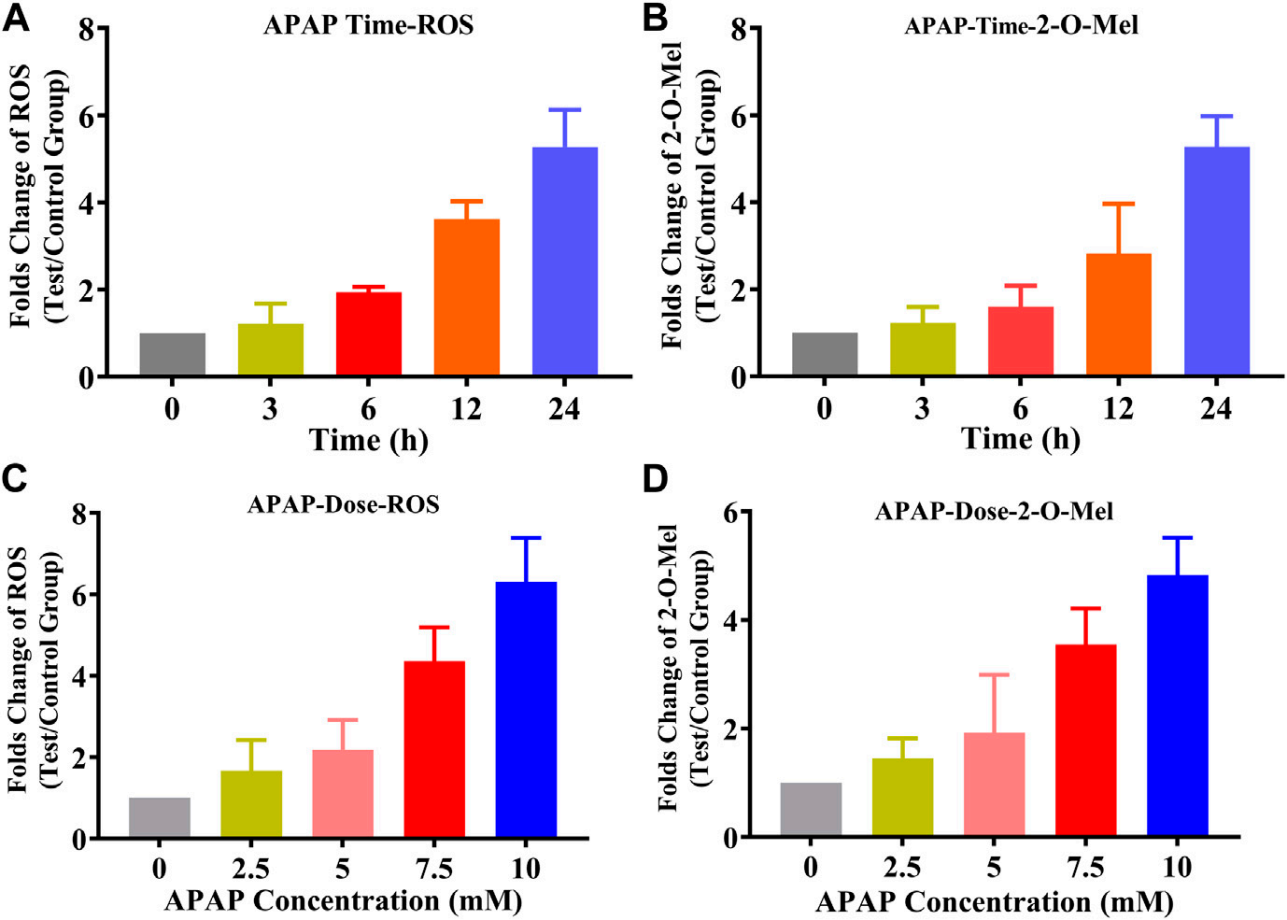
**(A)** Production of ROS with the time change (0–24 h) under APAP treatment (the measurement of ROS is assayed using a ROS assay kit (DCFH-DA)). **(B)** Production of 2-O-Mel with the time change (0–24 h) under APAP treatment. **(C)** Production of ROS with the time change (0–24 h) under APAP treatment. **(D)** Production of 2-O-Mel with an increase in the concentration of APAP.

In HepG2 cells, we established a model of reactive oxygen species (ROS) production induced by acetaminophen (APAP). As indicated in Figure 6, ROS production was measured using an ROS assay kit. The results demonstrated a dose-dependent (0–10 mM) and time-dependent (0–24 h) increase in ROS production in the presence of APAP. Alongside this, we quantitatively analyzed the formation of 2-O-Mel. As hypothesized, the production of 2-O-Mel in cancer cells in the presence of APAP mirrored the ROS levels, indicating both time and dose dependency. This evidence further supports the concept of ROS-mediated 2-O-Mel formation in cancer cells. These findings provide compelling evidence that ROS plays a key role in the formation of 2-O-Mel in cancer cells. Further research is required to explore the implications of this ROS-dependent metabolic pathway for cancer cell behavior and potential therapeutic strategies.

### 3.7 The mechanism of ROS-dependent formation of 2-O-Mel

**FIGURE 7:**
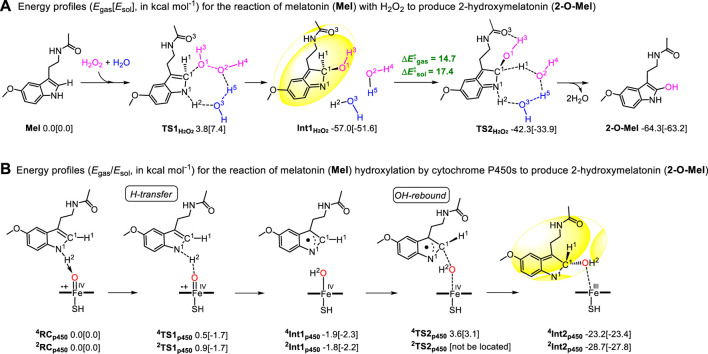
Energy profiles (*E*_gas_ (*E*_sol_), kcal mol^−1^) for **(A)** the reaction of melatonin (Mel) with H_2_O_2_ to produce 2-hydroxymelatonin (2-O-Mel) and **(B)** the hydroxylation reaction of melatonin (Mel) by the cytochrome P450 enzyme (Cpd I) to produce 2-hydroxymelatonin (2-O-Mel).

We experimentally observed this interesting phenomenon and explored the reaction mechanism of melatonin (Mel) with H_2_O_2_ to produce 2-hydroxymelatonin (2-O-Mel) by density functional theory (DFT) computations. The first step involves the O1‒H3 group of H_2_O_2_ attacking the carbon C1 atom of the Mel molecule. This forms a new C1‒O1H3 bond and transfers H2 (N1‒H2) to another O2-H4 of H_2_O_2_ through a transitional state, called TS1_H2O2_’ (Supplementary Figure S6). This reaction must overcome an energy barrier of 27.2 [26.5] kcal mol^−1^. This higher energy barrier prompted the search for an alternative route (Figure 7A) by examining if the H_2_O molecule could assist the transformation. To our delight, the substrate went through a lower transition state TS1_H2O2_ [3.8 (7.4) kcal mol^−1^] to generate an intermediate Int1_H2O2_ by an energy release of 57.0 [51.6] kcal mol^−1^. During this step, extra H_2_O aids in forming the C1–O1H3 bond and breaking the O1–O2/N1–H2 bonds. In reverse, the H proton (NBO charges: H1: 0.507, H2: 0.544, and H5: 0.576) deliveries generate the final product 2-O-Mel and two molecules of H_2_O. This change must surpass an energy barrier of 14.7 [17.4] kcal mol^−1^ (known as TS2_H2O2_) and release energy at a rate of 7.3 [11.7] mol^−1^. Comparatively, the process assisted by H_2_O is more favored and occurs more easily under the reaction conditions. Therefore, H_2_O plays an important role in the hydroxylation of melatonin. The geometric analysis indicated that one hydrogen-bond interaction (distance in 1.84 Å) between the H3 atom of -O1H3 and the O3 atom of -C=O exists in the transition state TS2_H2O2_. This additional interaction stabilizes TS2_H2O2_ and makes the hydroxylation of Mel accessible.

Furthermore, the hydroxylation reaction of melatonin *in vivo* was also studied by DFT computation. Numerous computational studies confirmed that the hydroxylation reaction generally occurs via two steps, viz., hydrogen abstraction and OH rebound (Meunier et al., 2004; Wang et al., 2015; Zhang et al., 2017). Combined with the aforementioned H_2_O_2_-participating process, two H-abstractions from C1‒H1 and N1‒H2 bonds were tried. However, the transition state of the former could not be located. The process was discovered to be endergonic by 25.6 [24.4] kcal mol^−1^ by directly optimizing the product (^4/2^Int1_p450_’) of H1 transfer (Supplementary Figure S6B). By contrast, the H2 (NBO charges: 0.245/0.248) transfer of N1‒H2 must overcome an extreme energy barrier (^4^TS1_p450_: 0.5 [‒1.7])/^2^TS1_p450_: 0.9 [‒1.7]) kcal mol−1 for HS/LS at the E_gas_/E_sol_ level (Figure 7B). This H transfer becomes a barrierless process in the bulk polar effect (Zhang et al., 2017). Therefore, the later H-abstraction from the N‒H bond is easier to take place than that from the C‒H bond. Further spin density (Supplementary Table S2) and NBO charge analyses showed that the NBO charges and spin densities of the H2 atom in transition states ^4^TS1_p450_/^2^TS1_p450_ are 0.245/0.248 and −0.01/0.00, respectively. However, the spin density distribution on the “substrate Mel” moiety in ^4^Int1_p450_/^2^Int1_p450_ approaches ±1. Therefore, this H-abstraction follows the proton-coupled electron transfer (PCET) (Mayer et al., 2002; Hirao and Chuanprasit, 2015; Li et al., 2015; Zhang et al., 2017). Starting from the intermediate ^4^Int1_p450_/^2^Int1_p450_, the OH atom rebounds to the C1 atom. This happens through an energy barrier of 5.5 [5.4] kcal mol^−1^ and results in a high spin state, releasing energy and forming the ^4^Int2_p450_ intermediate an energy release of 21.3 [21.1] kcal mol^−1^, whereas the transition state of OH-rebound for the doublet spin state was not located. This situation is coincided with the P450-catalyzed C–H bond hydroxylation reported; therein, the OH-rebound route often becomes barrierless on the LS state (Meunier et al., 2004; Wang et al., 2015; Zhang et al., 2017). In ^4^Int2_p450_/^2^Int2_p450_, a similar part (shadow) can be observed in Int1_H2O2_; therefore, we guess that the same H proton delivery occurs both in H_2_O_2_ and P450 systems. Furthermore, another transition state TS3_p450_ (Supplementary Figure S6C), as a TS2_H2O2_ chiral isomer, was optimized. Here also, it must overcome the same energy barrier of 14.7 [17.4] kcal mol^−1^. The optimized geometries for key transition states are shown in Supplementary Figure S7.

In conclusion, our DFT computations of the ROS-dependent formation of 2-O-Mel indicate that both water molecules and the –NHC = O group play a vital role in this hydroxylation reaction. The rate-controlling steps in both mentioned systems involve the hydrogen delivery assisted by two water molecules and an additional hydrogen-bond interaction. Our findings provide a more comprehensive understanding of the reaction mechanism of Mel with H_2_O_2_, which could be beneficial for future research on cancer metabolism and therapeutics.

### 3.8 The application of ROS-dependent formation of 2-O-Mel

**FIGURE 8:**
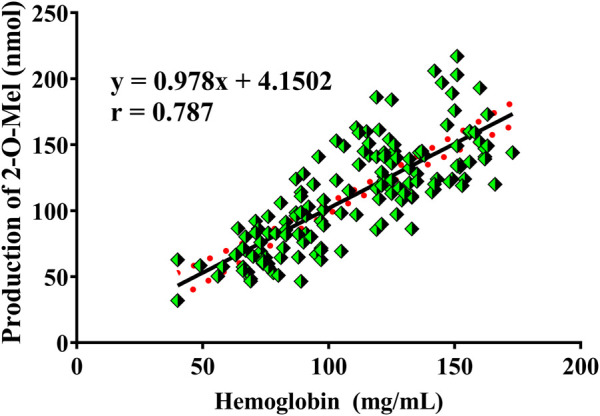
Correlation analysis between the production of 2-O-Mel in the plasma incubation system in the presence of Mel and the concentration of hemoglobin from 149 individuals *in clinics*.

In humans, hemoglobin is a vital function protein responsible for carrying oxygen in blood. Reactive oxygen is the major form of oxygen in hemoglobin. In this study, fresh blood was proved to possess the ability to catalyze the hydroxylation of Mel to generate 2-O-Mel. Thus, human blood from 149 healthy individuals was incubated with Mel, and the formation of 2-O-Mel was measured. The detailed method is described in Section 2.12. As shown in Figure 8, all the blood samples catalyzed the reaction of Mel hydroxylation to form 2-O-Mel, but the catalytic efficiency was significantly different. The results of routine blood test analysis showed a high correlation between the production of 2-O-Mel and hemoglobin concentration in individual blood samples, with the coefficient r-value being 0.787. In view of hemoglobin having the capacity of carrying reactive oxygen, our results indicated that the production of 2-O-Mel could reflect the oxygen-carrying capacity of hemoglobin to some extent.

## 4 Discussion

Mel, as an endogenous tryptophan-derived molecule, possesses powerful bioactivity in the progress of various diseases (Kennaway, 2019). In the previous study, the metabolism pathway of Mel in humans had been fully elucidated, which was mainly the enzyme-dependent metabolic progress. In our present study, the metabolism pathway of Mel in cancer cells was identified, seven novel metabolites were detected, and these findings further enriched the Mel metabolic network, especially in cancer cells. The metabolism heterogeneity in cancer cells indicated that the drug metabolism in cancer cells might be abnormal than in the liver and requires targeted research, and the metabolism progresses in cancer cells would provide some important guidance for the treatment. Free radicals and reactive oxygen species (ROS) are produced in the cells in enzymatic and non-enzymatic reactions (Zhang et al., 2016; Moloney and Cotter, 2018; Yang and Lian, 2020). Generally, Mel is also known as a ROS tracker that eliminates the ROS state; however, the ROS-dependent metabolism of Mel had not been fully illustrated, except for *N* ^1^-acetyl-*N* ^2^-formyl-5 methoxykynuramine (AFMK) and AMK production. Herein, 2-O-Mel, as a major ROS-dependent metabolite in cancer cells, was confirmed, which was another ROS-dependent elimination type, especially in cancer cells. Additionally, CYP450 also catalyzed the 2-O-Mel production, although they had low catalytic activity. It is worth noting that the metabolic heterogeneity of Mel between cancer cells and the liver or intestine tissues also suggested that there may be abnormalities in drug metabolism in cancer cells, which deserves further and detailed research. Additionally, the CYP450 enzyme-dependent 2-O-Mel production had also enriched, and the catalytic mechanism had also been illustrated. The geometrical optimization in the use of B3LYP/6–311+G (2d, p) was involved to provide a rational starting point for the under-studied ligand Mel when it comes to docking simulation. In addition, partial charges are important parameters when building the force field for the studied ligand Mel before MD simulation. In this case, we involve QM-level studies for such rational preparations. In one word, our results indicated that the ROS-dependent 2-O-Mel production is another pathway of Mel in clearing oxygen free radicals, especially in cancer cells. Finally, 2-O-Mel generation had been applied to measure the oxygen-carrying capability of hemoglobin. The obtained result indicated that the hemoglobin carried by oxygen was also a form of reactive oxygen (Zapora and Jarocka, 2013). All our findings not only enriched the metabolic pathway of Mel but also provided some useful guidance for the rational use of Mel, especially under the abnormal state (inflammation, cancer, acute infection, or other high ROS-intensive diseases) of our body in daily life.

## 5 Conclusion

In summary, the metabolic pathway of Mel in cancer cells was improved and enriched, and seven novel metabolites were discovered in cancer cells. Among them, 2-O-Mel proved to be the major metabolite style in cancer cells. Our results suggested that the generation of 2-O-Mel was ROS- and CYP1A1/2-dependent. Although the heme group in CYP450 participated in the enzyme-dependent catalytic reaction, quantitative analysis fully confirmed that ROS catalysis was the major source of 2-O-Mel in cancer cells. Moreover, the production of 2-O-Mel exhibited a good linear correlation with the concentration of hemoglobin, the active oxygen carrier in blood. Our results indicated that Mel also undergoes an extensive metabolism in the blood. Finally, the catalytic mechanisms of ROS and CYP450 toward Mel were fully elucidated by MD and DFT studies. The reactive oxygen species-mediated Mel conversing to 2-O-Mel not only provided a novel *in vitro* assay method to evaluate the oxygen-carrying capability of hemoglobin but also suggested ROS playing a significant role in the drug metabolism should be paid more attention.

## Data Availability

The datasets presented in this study can be found in online repositories. The names of the repository/repositories and accession number(s) can be found in the article/Supplementary Material.
