# Supplementary material for: ROS-dependent catalytic mechanism of melatonin metabolism and its application in the measurement of reactive oxygen
Source: Front Chem. 2024 Jan 16;11:1229199. doi: 10.3389/fchem.2023.1229199 (PMC10824942; doi:10.3389/fchem.2023.1229199)
Supplement: Supplementary file 1 [file DataSheet1.docx]

***Supporting Information***

**ROS-dependent Catalytic Mechanism of Melatonin Metabolism and Its Application in the Measurement of Reactive Oxygen**

Xiangge Tian ^1, 2+^, Xiaohui Kang ^3+^, Fei Yan ^1+^, Lei Feng ^1^, Xiaokui Huo ^1^, Houli Zhang ^3^, Yan Wang ^3^, Xia Lv ^3^, Xiaochi Ma ^1^, Jinsong Yuan ^2 *^, Jiao Peng ^2 *^ and Li Dai ^1, *^

^1^ Second Affiliated Hospital, Dalian Medical University, Dalian 116044, China.

^2^ Department of Pharmacy, Peking University Shenzhen Hospital, Shenzhen 518036, China.

^3^ College of Pharmacy, Dalian Medical University, Dalian 116044, China.

^+^ These authors contributed equally to this work.

***Corresponding authors**

**E-mail:** [yjs888@163.com](mailto:yjs888@163.com) (J. S. Yuan), pengjiao153@163.com (J. Peng) and daily21st@aliyun.com (L. Dai).


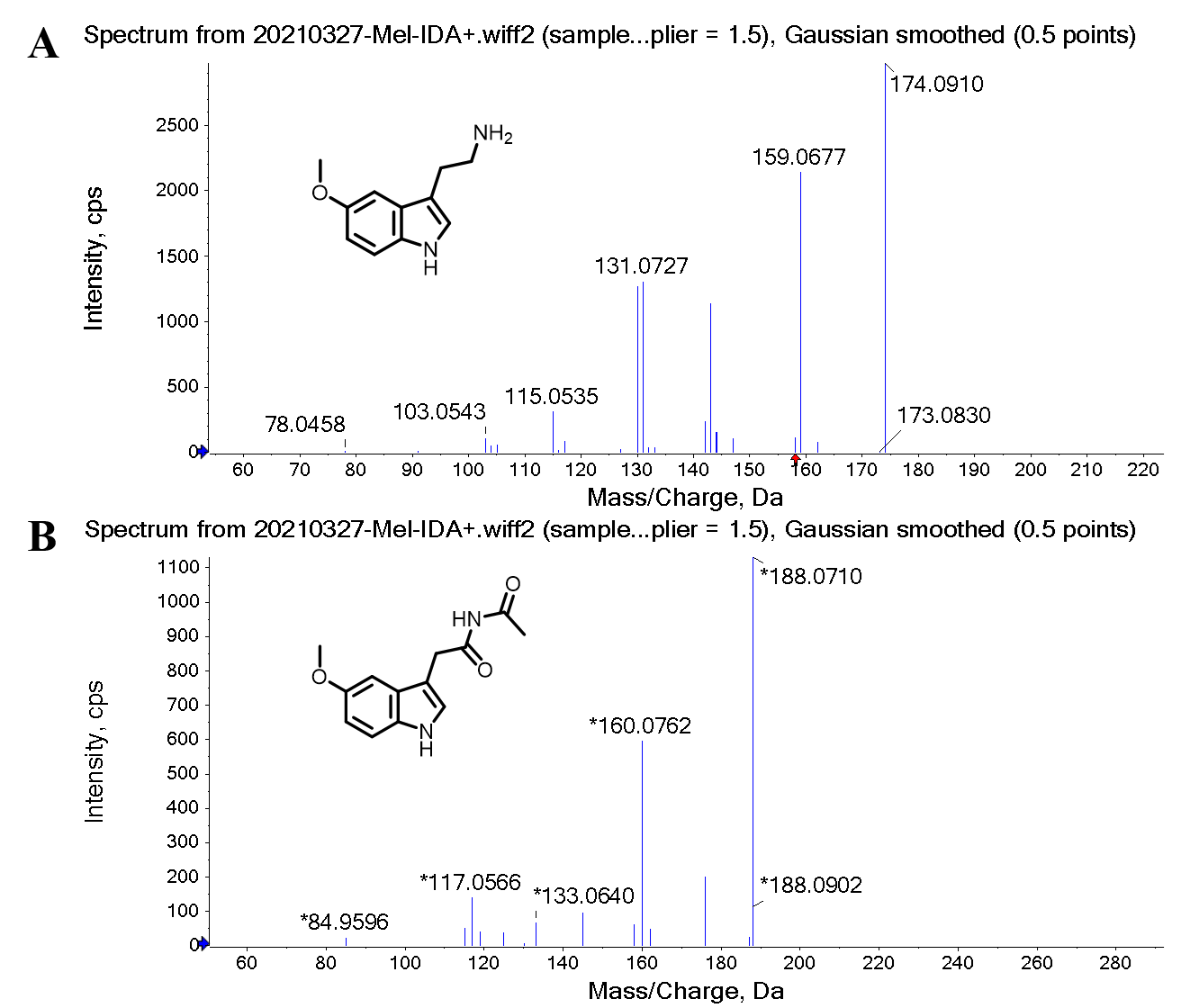


**Fig. S1** The TOF-MS/MS fragments for M4 (A) and M-10 (B).


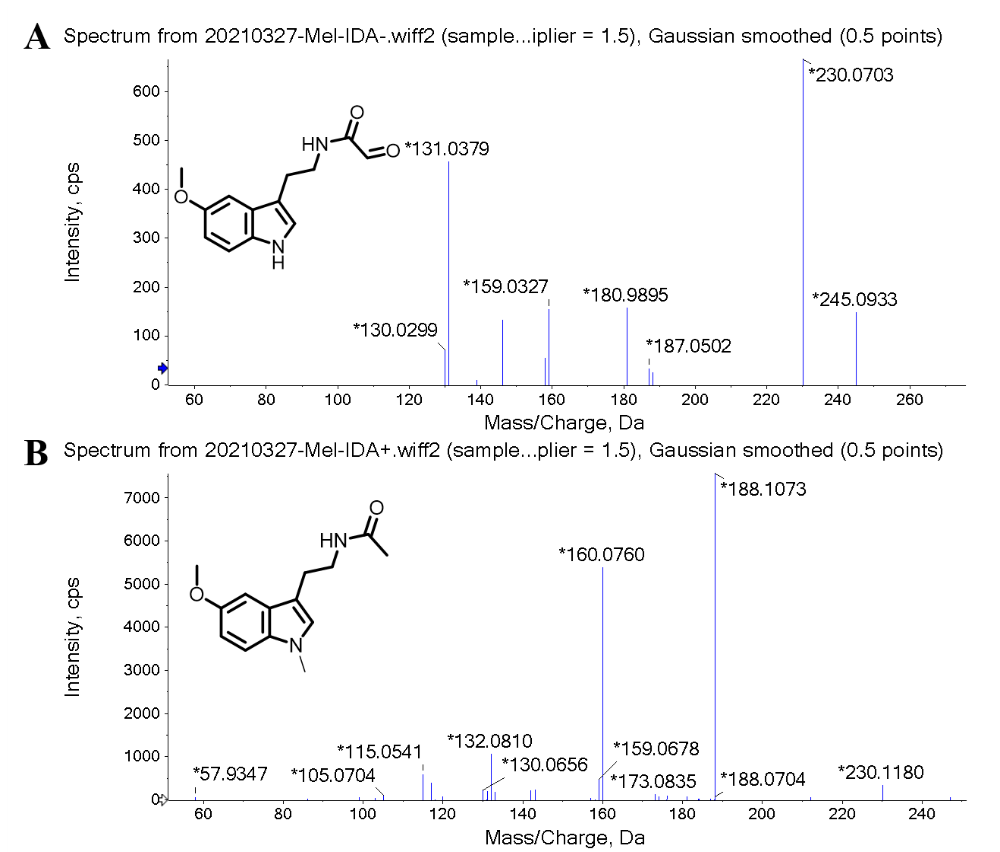


**Fig. S2** The TOF-MS/MS fragments for M11(A) and M-12(B).


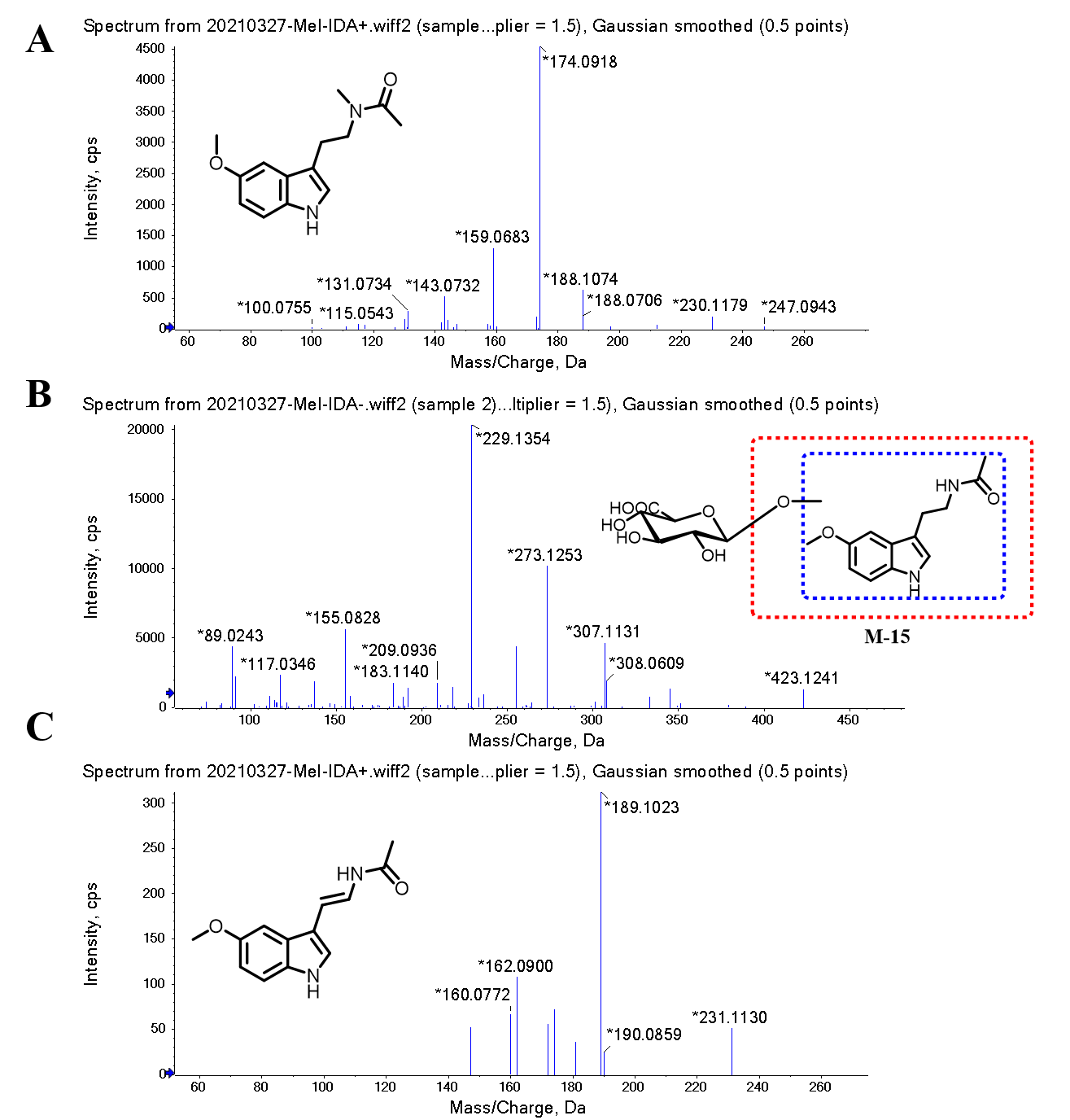


**Fig. S3** The TOF-MS/MS fragments for M13(A), M-15(B) and M-16(C).


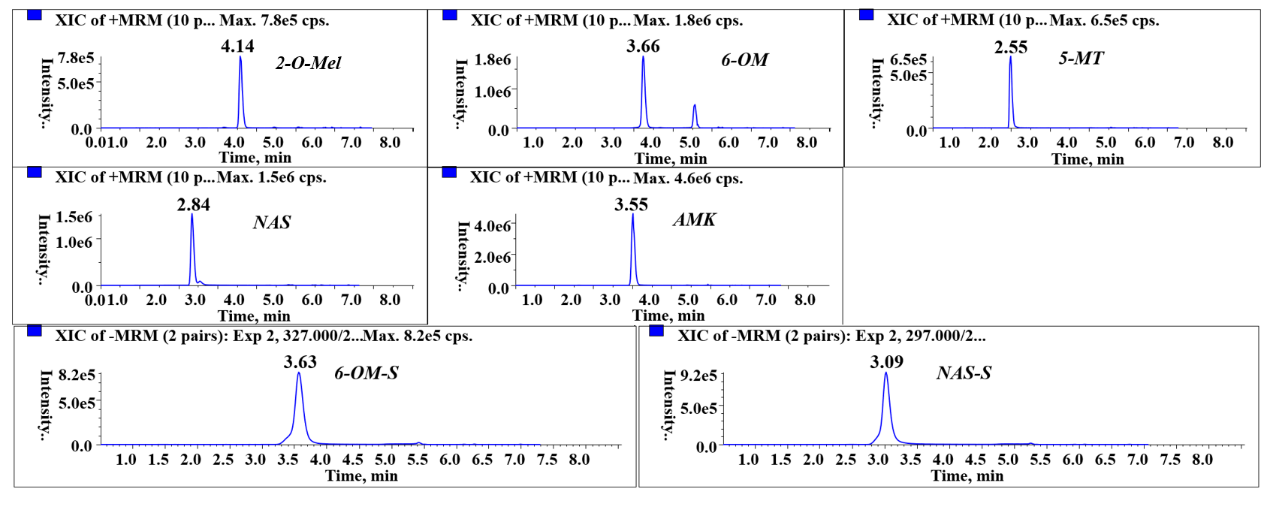


**Fig. S4** MRM chromatogram of 7 major metabolites of Melatonin.


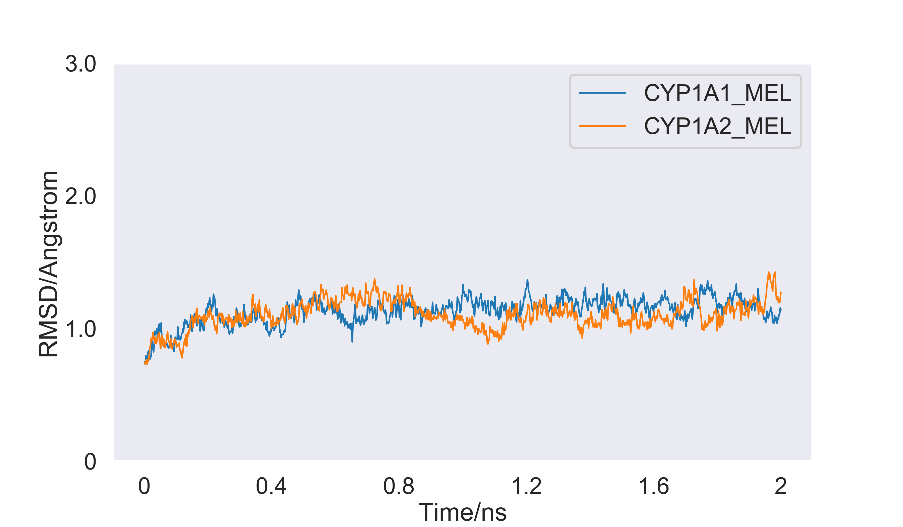


**Fig. S5** The system RMSD for molecular docking of Mel and CYP1A1 and CYP1A2.

**Fig. S6**. Energy profiles (*E*_gas_[*E*_sol_] for A) the reaction of melatonin and H_2_O_2_ without H_2_O, B) H-abstraction from C‒H bond, C) the N‒H bond formation and C‒H bond cleavage with the auxiliary of H_2_O.


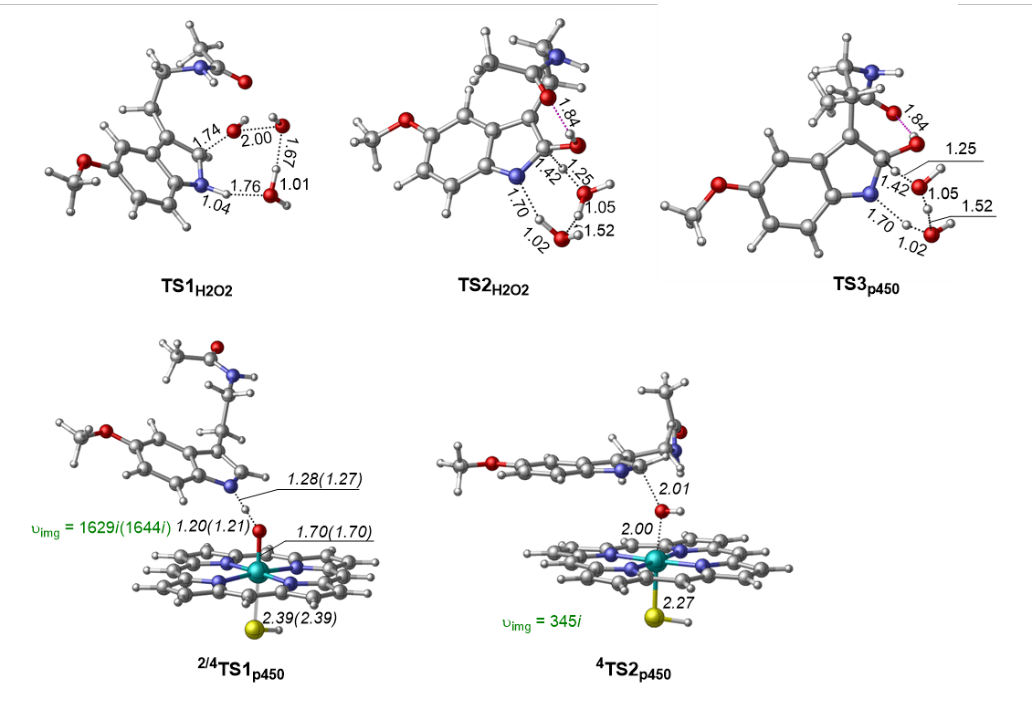


**Fig. S7.** Optimized geometries (distance in Å, vibrational frequencies in cm^-1^) for key transition states involved in Figure 7.

**Table S1**. The optimal *m/z* transition conditions for the metabolites analysis in LC-MS/MS.

|  | **Compounds** | **Q1** | **Q3** | **DP** | **EP** | **CE** | **CXP** |
| --- | --- | --- | --- | --- | --- | --- | --- |
| **Positive Model** | **2-O-Mel** | 249 | 162 | 80 | 9 | 36 | 11 |
|  | **6-OM** | 249 | 158 | 60 | 10 | 33 | 11 |
|  | **5-MT** | 191 | 174 | 80 | 10 | 15 | 13 |
|  | **NAS** | 219 | 160 | 72 | 10 | 20 | 15 |
|  | **AMK** | 237 | 114 | 40 | 12 | 14 | 15 |
| **Negative model** | **6-OM-S** | 327 | 247 | -90 | -10 | -27 | -15 |
|  | **NAS-S** | 297 | 217 | -90 | -10 | -35 | -15 |

**Table S2**. The Mulliken spin density of key reaction species for hydrogen abstraction and OH-rebound processes of **Mel** calculated at the B3LYP/BSI level.

| Species | Spin density | | | | | |
| --- | --- | --- | --- | --- | --- | --- |
|  | Fe | O | por | SH | H2 | rest |
| **^2^RC_p450_** | 1.30 | 0.80 | ‒0.53 | -0.53 | 0.00 | ‒0.04 |
| **^2^TS1_p450_** | 1.55 | 0.53 | ‒0.39 | ‒0.02 | 0.00 | ‒0.67 |
| **^2^Int1_p450_** | 1.72 | 0.30 | ‒0.12 | 0.10 | 0.01 | ‒1.00 |
| **^2^Int2_p450_** | 1.13 | 0.00 | 0.00 | ‒0.03 | 0.00 | ‒0.10 |
| **^4^RC_p450_** | 1.18 | 0.84 | 0.44 | 0.48 | 0.00 | 0.07 |
| **^4^TS1_p450_** | 1.49 | 0.54 | 0.14 | 0.12 | ‒0.01 | 0.73 |
| **^4^Int1_p450_** | 1.72 | 0.30 | ‒0.12 | 0.10 | 0.00 | 1.01 |
| **^4^TS2_p450_** | 0.97 | 0.32 | 0.11 | 0.14 | ‒0.01 | 1.47 |
| **^4^Int2_p450_** | 2.49 | 0.00 | 0.04 | 0.48 | 0.00 | ‒0.01 |
